# Towards achieving transnational research partnership equity: lessons from implementing adaptive platform trials in low- and middle-income countries

**DOI:** 10.12688/wellcomeopenres.18915.1

**Published:** 2023-03-16

**Authors:** Chelsea Modlin, Jeremy Sugarman, Gershom Chongwe, Nancy Kass, Winfred Nazziwa, Jemee Tegli, Prakriti Shrestha, Joseph Ali

**Affiliations:** 1 Berman Institute for Bioethics, Johns Hopkins University, Baltimore, MD, 21205, USA; 2 Division of Infectious Diseases, Johns Hopkins Medicine, Baltimore, Maryland, 21205, USA; 3 Division of General Internal Medicine, Johns Hopkins Medicine, Baltimore, Maryland, 21205, USA; 4 Department of Health Policy and Management, Johns Hopkins Bloomberg School of Public Health, Baltimore, Maryland, 21205, USA; 5 School of Public Health, University of Zambia, Lusaka, Zambia; 6 Uganda National Council for Science and Technology, Kampala, Uganda; 7 Family Health International Clinical/Partnership for Research on Vaccines and Infectious Diseases in Liberia, Monrovia, Liberia; 8 Department of International Health, Johns Hopkins Bloomberg School of Public Health, Baltimore, Maryland, 21205, USA

**Keywords:** international research partnership; equity; adaptive platform trials; adaptive clinical trials; public health emergencies

## Abstract

**Background:** Use of adaptive clinical trials, particularly adaptive platform trials, has grown exponentially in response to the coronavirus disease (COVID-19) pandemic. Implementation of these trials in low- and middle-income countries (LMICs) has been fostered through the formation or modification of transnational research partnerships, typically between research groups from LMICs and high-income countries (HICs). While these partnerships are important to promote collaboration and overcome the structural and economic disadvantages faced by LMIC health researchers, it is critical to focus attention on the multiple dimensions of partnership equity.

**Methods:** Based on informal literature reviews and meetings with leaders of one of the multinational COVID-19 adaptive platform trials, we describe what can be learned about research partnership equity from these experiences.

**Results:** We organize these considerations into eight thematic categories: 1) epistemic structures, 2) funding, 3) ethics oversight, 4) regulatory oversight, 5) leadership, 6) post-trial access to interventions, data, and specimens, 7) knowledge translation, and 8) research capacity strengthening and maintenance. Within each category we review the normative claims that support its relevance to research partnership equity followed by discussion of how adaptive platform trials highlight new dimensions, considerations, or challenges.

**Conclusion:** These observations provide insight into procedural and substantive equity-building measures within transnational global health research partnerships more broadly.

## Introduction

Recent infectious disease epidemics and pandemics have brought increased attention to the use of clinical trials that deploy adaptive designs. This term encompasses a wide range of trial designs that allow for predetermined opportunities to modify the protocol based on interim data analyses, and enact statistical and operational adjustments so the validity and integrity of the final trial results are maintained ^1, 2^. In particular, several large multinational adaptive platform trials were prominent contributors to global discussions and guidance formation for management of coronavirus disease (COVID-19) ^3^, including trials such as RECOVERY ^4– 6^, SOLIDARITY ^7^, and Randomised, Embedded, Multi-factorial, Adaptive Platform Trial for Community-Acquired Pneumonia (REMAP-CAP) ^8^. Adaptive platform trials compare multiple interventions against one another, commonly sharing a singular control group and adding or dropping intervention arms based on predefined decision points and cumulative data evaluation. In the context of an epidemic or pandemic where countless lives are at risk, there have been arguments that favor this type of trial design on both operational grounds (large-scale clinical trials with conclusive results emerging more quickly to dictate clinical practice) and ethical grounds (minimizing the number of participants randomized to control arms and boosting the anticipated benefit to risk ratio given ability to add or drop interventions based on efficacy and safety). Adaptive platform trials are also viewed by some as being more ‘synergistic’ with clinical management by providing a panel of potential therapeutic options as is done in clinical medicine, minimizing the chances of a participant receiving no intervention, and being more adept at responding to constantly evolving medical knowledge, clinical care, and public policy shifts during outbreak response ^9^.

 In response to the COVID-19 pandemic, adaptive platform trials were rapidly implemented in the United States and Western Europe ^10, 11^. As the pandemic progressed, the need for low- and middle-income country (LMIC) ^1^ inclusion became evident. Several adaptive platform trials began fostering research partnerships and incorporating LMIC study sites as they had for other outbreaks ( Table 1), such as the Ebola virus. Transnational partnership between two or more international collaborators is a common model for externally funded health research in LMICs, including for recent clinical platform trials using adaptive designs. While partnerships between two or more LMICs are very important ^12^, many if not most international clinical trial partnerships are between high-income countries (HICs) and LMICs ^13, 14^. HIC partners ^2^ often contribute financial resources and scientific experience, to complement LMIC partners’ own resources, scientific capabilities and experiences, social capital, and understanding of the local health and health research landscape. These types of partnerships are prioritized as one of the United Nations’ Sustainable Development Goals (SDGs) ^15^ and equity between partners is considered essential for ethical partnership-based research in LMICs ^16^. Equity is a foundational principle of the conduct and outcomes of global health research ^17^ with justification for its application in transnational research partnerships grounded in theories of global justice ^18, 19^.

**Table 1.** Examples of platform adaptive clinical trials involving low- and middle-income countries (LMICs).

Type of adaptive design	Description	Examples	LMIC involvement
Multiarm multistage (MAMS)	Compares multiple experimental groups against a shared control group. Permits early stopping of non- promising or highly effective arms and/or initiation of new arms using prespecified criteria.	RECOVERY (coronavirus disease [COVID-19])	Ghana, India, Indonesia, Nepal, Vietnam
		SOLIDARITY (COVID-19)	Bangladesh, Bolivia, Egypt, Ethiopia, Honduras, India, Indonesia, Iran, Kenya, Lebanon, Mali, Mozambique, Niger, Nigeria, Pakistan, Philippines, Sierra Leone, Zimbabwe
		CROWN-CORONATION (COVID-19)	Ghana, Zambia
		TB-PRACTECAL (Tuberculosis)	Uzbekistan
		TRUNCATE-TB (Tuberculosis)	Indonesia, Philippines
		PALM (Ebola)	Democratic Republic of the Congo
Response-adaptive randomization (RAR)	Allows for changes to the randomization probabilities based on interim analysis of ongoing trial results.	PREVAIL II (Ebola)	Liberia, Sierra Leone, Guinea
		REMAP-CAP (COVID-19)	Pakistan, India, Nepal

However, these partnerships are not without challenges. In many LMICs, it can be particularly difficult to acquire and produce scientific knowledge due to historical, structural, economic and geopolitical influences that impose disproportionate capability limitations. Imbalances and differences in terms of partner experience, research interests, institutional support, and access to financial and material resources ^20^ – among other considerations – risks inequitable and suboptimal outcomes of the partnership. This is compounded by the fact that some of these imbalances can be rooted in the historical colonialization of many LMICs, with contemporary structures echoing systematic oppression and maintaining barriers to intellectual and scientific leadership among LMIC investigators and research groups ^21^. These asymmetries can be directly or indirectly reflected in organizational contracts and memoranda of understanding used to form and maintain research partnerships, influence allocation of research funding, impact the availability of local scientific mentorship and methodological training for core capacity strengthening efforts, and further perpetuate asymmetry in research partnership outputs.

There is widespread acknowledgement of research partnership inequities ^22– 24^ and dedicated efforts – such as the Research Fairness Initiative led by the Council on Health Research and Development ^25^ and the Equity Tool for valuing Global Health Partnerships developed by the Canadian Association for Global Health ^26^ – to evaluate equitability of collaborating partners. There is a growing literature of qualitative studies contextualizing recurrent themes within research partnership equity ^27– 29^ and scoping reviews of guidance frameworks suggest we are approaching a consensus around the major themes that contribute to research partnership equity ^30, 31^. Yet the operationalization of equity recommendations from principle to practice in research partnerships remains poorly understood and has largely been restricted to descriptions of program practices ^32^, anecdotal commentaries and opinions, condemning ‘parachute’ or ‘parasitic’ research practices ^22, 33^, or limited to discussions about authorship ^34– 36^ and bibliographic trends ^37^. While these efforts are necessary and important, they are not sufficient to claim that the complexities around research partnership inequities are fully understood nor have optimal mechanisms for preventing them been identified.

In this report, we describe what can be learned about research partnership equity from the experiences of implementing multinational adaptive clinical trials during the COVID-19 and Ebola pandemics with a particular emphasis on adaptive platform trials. We offer a thematic analysis of eight areas where asymmetries exist within transnational research partnerships: epistemic structures, funding, ethics oversight, regulatory oversight, leadership, post-trial access to interventions, data, and specimens, knowledge translation, and research capacity strengthening and maintenance. For each area, we start with discussion of general considerations including the normative claims that have been offered previously in efforts to promote equity in transnational research partnerships. This is followed by some of the unique ways in which adaptive platform trials contribute their own complexities and potential solutions to issues within research partnership equity. These issues are important for all types of global health collaborations. While not unique to adaptive trials, they become heightened because of the coordinated multinational reach, dynamic adaptability of research objectives, and substantial investment in scientific infrastructure that are associated with adaptive platform trials in particular. We emphasize how the implementation of these trials can highlight opportunities to narrow equity-related asymmetries between LMIC and HIC research partners. 

## Methods

This analysis was produced as a part of commissioned project conducted with support from the World Health Organization (WHO) Ethics and Governance Unit focusing on ethics and adaptive clinical trial designs in public health emergencies. Given the constraints of the project timeline and broad scope of the research questions, a full systematic review of the literature was not conducted. Rather, we performed a rapid narrative review ^38^ on transnational research partnership equity, which was cross referenced with selective reviews of literature on adaptive clinical trial designs and adaptive platform designs, including those reporting COVID-19 and Ebola research. This approach allowed for broad examination of emerging evidence, evaluation for more specific lines of inquiry, clarification of concepts, and synthesis of two largely separate but theoretically converging lines of published literature.

The PubMed/MEDLINE, Scopus, and Google Scholar databases were reviewed in March and revisited in May of 2022 by authors CM and PS using keyword searches for ‘equity’ OR ‘fairness’ AND ‘international research partnerships’ OR ‘global research partnerships’ ‘OR ‘transnational research partnerships’ OR ‘North-South partnerships.’ The selective literature search on adaptive clinical trials focused on ethical analyses and operationalization of these trial designs and included search terms ‘adaptive clinical trial +/- designs’ OR ‘platform trial’ AND ‘ethics’ OR ‘ethical considerations’ OR ‘experience’ OR ‘operationalization.’ The references of reviewed publications were appraised, and relevant citations were included in subsequent abstract and full-text reviews. Each author extracted themes and topics independently and then compared and discussed with a third author, JA, to reach consensus and minimize bias.

Informal virtual meetings between project participants (CM, PS, and JA) and leadership teams of REMAP-CAP were held via zoom in April 2022 to enhance understanding of the literature, triangulate themes, and identify some unpublished experiences and practices. A preliminary draft of this report was then discussed by authors CM and JA during a project group meeting in Geneva, Switzerland in July 2022. Findings were iteratively revised based on feedback from this meeting and from critical review by experts in ethics, global health, and adaptive clinical trial methods (authors JS, GC, NK, WN, and JT). In what follows, findings are presented according to eight thematic areas ( Table 2) that were deductively derived from the literature review and meetings. Each theme begins with a general review of the topic, including discussions of implications for international research partnership equity. In each section, we then offer additional considerations that relate to trial partnerships that deploy adaptive clinical trial designs with a particular focus on multinational adaptive platform trials.

**Table 2.** Summary of themes and key points.

Theme	Key Points
Epistemic structures	• Includes both (1) access to existing systems of scientific publication and work that informs the relevance, importance, and design of a partnership’s objectives and research plan, and (2) influence over how knowledge is generated and produced within and by the partnership. • High-income country (HIC) partners likely have disproportionate adaptive trial methodological expertise within a transnational partnership, this is one of the inherent imbalances that needs to be accounted for when determining goals for the partnership, concordance with local research objectives, and low- and middle-income countries (LMIC) research capacity.
Funding	• Prior to the coronavirus disease (COVID-19) pandemic, global health funding mechanisms were less familiar with adaptive platform trials and the budgets were not always sufficient for complex study implementation requirements. This limited the feasibility of conducting adaptive trials overall, but particularly within LMICs. • Significant up-front investment in adaptive trials can provide a certain degree of financial security provided there is dedicated time and effort to incorporate domestic and other sustainable mechanisms of funding long-term.
Ethics Oversight	• Despite major improvements in research ethics committee (REC) and data monitoring committee (DMC) capacity, infrastructure and resources for ethical oversight remain underdeveloped in many LMIC contexts. • Partnering with or providing support to LMIC RECs/DMCs would help with the increased burdens associated with oversight of large research partnerships, especially during public health emergencies. • There is a need for REC/DMC familiarity with adaptive platform trial designs, including unique ethical considerations of these different methodologies, as this may not fall under the current expertise of many RECs/ DMCs.
Regulatory Oversight	• Regulatory oversight requires a careful balance of mechanisms that protect LMIC research participants and communities from exploitation without imposing insurmountable restrictions on research partnerships and institutions. • LMIC-specific guidance for the regulatory oversight of multinational adaptive trials is needed as current guidance is developed mostly for HIC-only contexts.
Leadership	• Leadership can take many different forms, and within a research partnership there needs to be acknowledgement of the different strengths leaders from LMICs versus HIC can contribute. • Concentration of HIC leadership within adaptive trials can be avoided by early inclusion of LMIC partners in the study simulations and design and rotating distributive leadership positions.
Post-Trial Access,	• Research resource sharing should be bidirectional and ought not preclude LMIC access to data and specimens. • Post-trial access to interventions is a complex issue and, at minimum, must be negotiated early during the research process and be mutually agreed upon by both LMIC and HIC partners. • Post-trial access to interventions within adaptive platform trials is complicated by the dynamic nature of the research design.
Knowledge Translation	• LMIC guidance on the translation of research findings into policy is critical. • Adaptive platform trials necessitate clear explanations for how the research design may have impacted interpretation of the results should be available for implementers and policymakers.
Research Capacity Strengthening and Maintenance	• Equity within transnational research partnerships requires dedicated attention to capacity strengthening to support developing research capabilities and processes. • The sizable financial and research training investment required to establish a large, adaptive platform trial may be uniquely positioned to provide built-in protections for research capacity strengthening efforts, provided there is ongoing attention to sustainability and seeking alternative or domestic longitudinal funding.

## Results

### Epistemic structures

Production of valid scientific results requires not only the resources to generate new knowledge, but also the means to access epistemic structures (i.e., how knowledge is defined, acquired, and categorized) including systems of scientific publication and dissemination of research. Despite recent growth in open-access publication ^39^, much of the relevant global health data and scientific literature remains concentrated in HIC settings with LMIC researchers often dependent on global health organizations, HIC sponsors, or HIC research collaborators to access these knowledge bases. There is a risk of defaulting to HIC-centric conceptions of what constitutes meaningful data and health outcomes without necessarily recognizing the extent to which culture, language, and context can influence meaning ^40^.

In terms of knowledge production within a research partnership, LMIC researchers are sometimes limited to the role of ‘data collectors,’ responsible for assembling samples and engaging in research fieldwork ^27, 41^. HIC researchers often have a stronger voice in determining who conducts and verifies analyses, and what is included in final products for dissemination. While these are all essential parts of the scientific process, this dichotomization between partner responsibilities not only underutilizes existing skill sets, but also limits LMIC researchers’ participation in activities that contribute to study design and interpretation of data ^42^ when integration of LMIC perspectives actually provide more validity to the work. This is reflected in an evaluation of international randomized clinical trials which found that across 305 clinical trials, data flowed exclusively from collection in LMICs to analysis in HICs for 73% of studies ^43^.

Clear expectations about the generation of research data and knowledge derivation should be outlined explicitly by partnerships in contracts or memorandums of understanding and reinforced by the institutional and financial sponsors who are supporting the research. If there are limitations to a partner’s ability to contribute to the design, synthesis, and analysis of the data, this should prompt investigation into necessary infrastructure or logistical support to narrow this divide and, when possible, be included in funding allocation and sponsorship. This includes investment in the professional development of LMIC researchers in relevant areas that contribute to research design and implementation ^44, 45^. This funding mission and structure is utilized by the Fogarty International Center at the National Institutes of Health (NIH) in the United States. While unlikely to address all needs, sometimes there are also external resources available that can be incorporated or leveraged by a research partnership, for example the WHO’s HINARI and Research4Life platforms that provide journal and literature access to low-income country institutions at low or no cost.

With respect to adaptive platform trials, a systematic review of master protocols found growing uptake of these designs worldwide; however, their use has been almost exclusively restricted to HICs ^46^. It follows that the majority of experience in the methodological design and conduct of adaptive platform trials to-date likely resides within HIC investigators and institutions. If this holds and HIC partners have disproportionate methodological expertise within a transnational adaptive trial partnership, this provides an opportunity for partnerships to orient some of their goals towards increasing methodological capacity of LMIC researchers.

LMICs were initially not included in the majority of multi-national clinical trials established early during the COVID-19 pandemic. Adaptive platform trials investigating a wide range of therapeutics rapidly started across thousands of sites in the United Kingdom, European Union and United States where the study designs and protocols for these trials were developed and implemented. Eventual inclusion of international sites, including in LMICs, required a careful balance. On one hand there are the universal objectives of a centralized research protocol and the need for data generated at newly added sites to be compatible and comparable to the rest of the study (i.e., a universal protocol). On the other hand, attention to the objectives and interests of LMICs and other international researchers and communities needs to be respected and incorporated (i.e. a more pluralistic approach to the needs of different populations). This includes pragmatic assessment of what therapies and interventions are or are likely to become available within a particular LMIC, which likely vary when compared to interventions in HICs. Additionally, while research data may need to be aggregated for some of the primary analyses in a multinational trial, local data should ideally be available for independent analysis by LMIC researchers for specific trends or findings that may impact local practice as would be true for any country. One of the benefits of adaptive platform trial designs is that addition or removal of study arms according to feasibility or needs of specific LMIC settings is possible. The debates about universal vs. plural approaches to global health research are not new ^47^. Growing experience with multinational adaptive designs is an opportunity to provide further empirical evidence about how these approaches influence the design and implementation of research.

 REMAP-CAP provides a helpful example ^8, 48– 50^. The roll out of REMAP-CAP in several LMICs in Asia was facilitated by partnering with an existing research network, the Collaboration for Research, Implementation and Training in Asia (CCA), which had overlapping research interests and allowed them to operationalize trial arms in India, Nepal, and Pakistan ^48^. Development of mutual goals and outcomes was enabled by establishing a LMIC-led local governance body to interface between REMAP-CAP leadership and local LMIC study sites. This helped to support early identification of feasible interventions and incorporation of LMIC investigators into the international steering committee, both of which allowed for integration of LMIC perspectives and input at multiple levels of the multinational platform trial.

### Funding mechanisms

Much of the power asymmetry between HIC and LMIC partners is believed to be rooted in access to funding ^51, 52^ and inequities that lead to writing a competitive grant proposal. The research agenda and priorities of a transnational partnership are frequently indirectly driven by HIC partner interests or dictated by funders based in HICs seeking global collective insight and scientific advancement. Typical processes for setting research agendas can overlook or underestimate pressing but less universal LMIC-specific needs ^53, 54^ and inadequately address the complex societal influences that disproportionately contribute to poor health outcomes in LMICs such as poverty, food insecurity, poor sanitation, and limited healthcare infrastructure. Major donors in both the public and private sectors are more likely to fund HIC researchers and, while increasingly interested in health equity, have been relatively absent from conversations about promoting research partnership equity ^55^. This results in LMIC researchers reporting the need to compromise on interest or relevance in order to access personal and research-related financial support ^27^. HIC partners are very often approached first to weigh in on how to structure a partnership, including assigning group-level responsibilities, establishing an activity timeline, and allotting research resources. As described by Kalinga ^56^ “Those who hold the purses dictate the terms.”

Some partnership models have challenged this archetype. The Ghanaian-Dutch Health Research for Development Programme (HRDP) ran from 2001–2008 in response to concern that the research priorities of LMICs were not being supported through traditional funding mechanisms. The Dutch government collaborated with Ghanaian researchers and showed that supporting demand-derived, LMIC-led research resulted in products that were likely to be implemented and used to inform health policy in Ghana. Partnerships with Netherlands-based researchers did occur in some instances as part of this collaboration, but the role of these HIC partners was described more as one of allyship and support to LMIC partners than direct involvement and leadership ^57^.

Because of the close relationship between prominent funding models and setting the research agenda, advocating for the interests and needs of LMIC communities may seem to be beyond the ability of individual HIC and LMIC partners to address. This speaks to the importance of working with funders of global health research to include diverse LMIC perspectives during the earliest stages of establishing research networks and priorities, and then making transparent how this involvement was incorporated and what it yielded. Justification for priorities decided upon need to be clearly outlined by a funder and endorsed by LMIC leadership and health officials. A careful balance is required, however, with expectations about duration and contributions of international funding for LMIC health priorities and how they factor into long-term sustainability and promotion of local funding mechanisms. The HRDP came to an end in 2008 after sponsorship support for the program from the Netherlands government ended. According to interviews with officials from Ghana’s Ministry of Health, an increase in research support from the Ghanaian government was not felt to be necessary to replace this lost investment to their research infrastructure because of the perceived accessibility of other international funding ^57^.

Funding for adaptive trials is susceptible to the same issues of funding global health research more generally. Prior to COVID-19 and Ebola, traditional global health funding mechanisms were less familiar with adaptive platform trials and the budgets awarded were not always sufficient to meet the needs of very large research networks and associated complex protocol development and study implementation requirements ^58^. This limited the feasibility of conducting adaptive trials overall, but especially in LMICs ^46^. All of this has changed with COVID-19, but the long-term ramifications of this shift in interest and funding allotment have yet to be fully actualized. Early dedicated funding to the initiation of these large trials led to their rapid scale up in HICs, partially driven by priorities of the funders to form study sites and enroll participants from the funder’s own country ^9^.

However, these are complex trials to design and implement and they require significant up-front investment from industry and other clinical trial sponsors. This degree of financial commitment can protect against the risks that come with unpredictable funding, such as compromise in research outcomes or falling short of projected research capacity strengthening efforts. This assumes, however, that LMIC partners are included in financial deliberations and allocation discussions from the beginning of the research process and design. Early, large financial commitments - as are often seen in adaptive platform trials - allows for more time to incorporate domestic and other more sustainable mechanisms for long-term funding.

### Ethics oversight

While some LMICs have robust mechanisms for local ethics and regulatory oversight of research ^59^, oversight infrastructure and resources remain underdeveloped in many LMICs, relative to HICs ^60^. There have been major efforts and improvements in research ethics committee (REC) and data monitoring committee (DMC) capacity ^61, 62^ to address the growing scope of ethical concerns around health research in LMICs. In addition, there has been increasing attention to the role of community advisory boards (for further discussion on this, please see *Davies et al.* in this series).

Research sponsors and institutions, especially those based in HICs, should consider providing direct support to LMIC RECs to help with the increased burdens associated with oversight of large research partnerships, especially during public health emergencies ^63^. This could include fostering partnerships between committees to help provide mutual support and perhaps even collaborative review of research proposals ^64^. Such partnerships could be between HIC and LMIC RECs, or solely between LMIC RECs, depending on where the relevant experience is located. There have been previous efforts to provide funding for international joint REC review. An example of this type of collaboration includes the joint scientific reviews by the WHO-African Vaccine Regulatory Forum (AVAREF) which brought together multidisciplinary teams of experts to review multinational vaccine trials during infectious disease outbreaks that shortened protocol review timelines and supported LMIC REC capacity strengthening ^65^. Similarly, the Global Emerging Pathogens Treatment (GET) consortium, which engages multidisciplinary African leaders in surveilling and responding to local and national public health emergencies, published its deliberations and approach to ethical review around the Ebola response, establishing an African-led framework for future epidemics ^66^.

Information on the role of DMCs in transnational research partnerships is sparse ^67^, including how often the composition of monitoring committees reflects the LMIC settings where the research is taking place. Given the methodology of adaptive trials with protocol adjustments based on interim data analyses, DMCs play a central role to their functioning and success. The expansion of adaptive trials into LMICs provides an opportunity to evaluate the structure and functioning of DMCs and, if needed, reassess how best to configure ethical and research oversight where LMIC inclusion and representation is essential.

There is a need for more familiarity with adaptive platform trial designs amongst RECs and DMCs, including unique ethical considerations of these different methodologies ^68^. This may not fall under the current expertise of many committees, particularly in LMICs where adaptive trials are a recent development. This issue is not isolated to LMICs, however. Delays associated with ethical review of adaptive trials have been noted in the European Union as well ^49^. Appropriate training and guidance to RECs and DMCs on evaluating protocols and justifications for adaptive platform trials is an important part of building local research capacity and helps avoid rejection of scientifically valid research based on methodological unfamiliarity ^69^.

### Regulatory oversight

Regulatory oversight in transnational research is highly variable and has been criticized in some situations as being too restrictive with the use of a universal or ‘one-size-fits-all’ approach to research monitoring without appreciating the nuances and complexities that come with different research study sites and resources ^70^. For some LMIC research regulators, motivating factors for imposing local restrictions and requirements have included general concerns about ‘outsourcing’ clinical research that would traditionally be regulatorily challenging to pursue in HICs ^71, 72^. While research participant protection has arguably been a target of these efforts, some regulatory actions have come at the cost of making research difficult to achieve. For example, in India ^73^ and Chile ^74^, the number of operating clinical trials decreased after requirements related to compensation for overly broad definitions of trial-related harm were imposed.

It is noteworthy that when seemingly burdensome regulatory requirements emerge within a small number of jurisdictions, even if they are well-justified, those who have control over global health research resources may simply elect in the present or future to fund research conducted elsewhere. For some, such an ability offers a paradigmatic reflection of the inequitable distribution of power between those who fund research and those who may benefit from its outputs. It also leads some to perceive global health research as something that operates in a privileged space between sovereign nations. That noted, national regulators also bear responsibility to consider the implications for citizens of blunt research oversight policy actions. Ultimately, there remains an acknowledged ^75^ but under-addressed need for those involved in funding, conducting research, and research oversight across multinational contexts to engage in substantive discussions about equitable research oversight. For example, re-evaluating processes for recognizing the legitimacy of alternative regulatory frameworks ^74^.

Adding complexity to the oversight context, large multinational platform adaptive trials typically have multiple sponsors form across different countries, who may or may not have a history of co-funding research. Yet, the International Conference of Harmonisation Good Clinical Practice Guidelines (ICH-GCP), which among other things enumerates sponsor responsibilities, are most frequently applied to research involving one study sponsor. Large, multinational platform trials that are well- and variably-resourced to address an epidemic or pandemic can make accountability and responsibility more confusing for both sponsors and regulators who may not have the experience or frameworks to coordinate with other sponsors or fulfil their roles in an organized manner ^9^. Basic regulatory and design requirements for clinical trials using adaptive methods are available from the U.S. Food and Drug Administration (FDA) ^76^ and the European Medicines Agency (EMA) ^77^. While this guidance helps standardize trial design and approach, these are recommendations based on experiences and regulatory bodies situated in HICs. Special regulatory considerations for internationally funded adaptive trials conducted in LMICs, and LMIC-specific guidance for these trials is needed.

### Leadership

Leadership can default to the HIC partner who typically has greater access to financial, training and material resources. Attempts to incorporate LMIC leadership can be perceived as tokenistic if relevant roles and responsibilities are not clearly discussed and delineated in formal research partnership agreements and actions and with an eye toward equity. Within a transnational research partnership there needs to be mutual leadership from all partners and acknowledgement of the different strengths leaders from each setting can contribute.

Within more localized and LMIC-specific epidemics, there is even more reason to ensure leadership is representative of the local research context. An example of this is the Partnership for Research on Ebola Virus in Liberia (PREVAIL). In 2014, discussions initiated by the Liberian minister of health ^78^ were held with the National Institutes of Allergy and Infectious Diseases (NIAID) in the United States who funded and formed PREVAIL to assess experimental interventions in response to the Ebola epidemic in Liberia. This partnership has subsequently evaluated two vaccine candidates for prevention (PREVAIL I) ^79^ and designed an adaptive study protocol to evaluate potential treatments (PREVAIL II) ^80^ among other Ebola-related studies ^81, 82^. Within these studies, LMIC leadership was recognized as making highly important contributions to the social mobilization and communication of Ebola prevention, including the formation of community-based task forces and support for individuals isolated under quarantine ^78^.

Leadership and experience with adaptive trials, especially those addressing global issues like COVID-19, has the potential to be concentrated to HICs where the majority of the centralized protocols are initially developed. This can be mitigated by early inclusion of LMIC partners in the study simulations and design. Because of their wide distribution and geographic range, it has been recommended that leadership within multinational adaptive trials not be isolated to one or a few individuals but be viewed more as ‘distributive leadership’ with a rotating steering committee and a separate intervention prioritization committee so power, influence, and decision-making are not concentrated within a small group of individuals ^9^.

## Post-trial access (PTA) to interventions, data, and specimens

Discussions around post-trial access are often delineated between access of study participants and the community to study-related interventions and access between research partners to study data and specimens. Both are relevant to research partnership equity and adaptive trial design. The terms of PTA to study data, lab specimens, and the interventions under investigation are ideally delineated prior to onset of collaborative trials in LMICs. This is admittedly a complex process and PTA access plans can sometimes be impossible to implement, particularly for study interventions ^83^. Frequently, there are local expectations of access to these resources; yet, more often than not continual access is challenging for LMIC partners ^84, 85^.

Post-trial access to interventions under investigation (if found to be beneficial) is a long-standing issue in global health research and often is viewed as a matter of global distributive justice ^18^, requiring input from stakeholders beyond a research partnership including local health officials and policymakers. International and national guidelines for post-trial access vary in their recommendations, stances on necessity, and details of how post-trial access should be made available ^86^. A research partnership’s obligations to supply or provide PTA to effective interventions must be negotiated early during the research process and be mutually agreed upon by both LMIC and HIC partners. While this is necessary, it may remain insufficient and further specifics within a partnership for post-trial access and responsibilities, such as transparency with research participants should be incorporated into the study protocol and informed consent processes. Providing post-trial access may be more realistic for some types of research interventions and partnerships over others, and some LMIC research partners may prioritize additional or even other types of study benefits, such as investments in scientific capacity and infrastructure. Regardless, this issue needs to be intentionally addressed within the partnership, with the community of research participants, and with local health policy makers to ensure potential direct or indirect research benefits align with LMIC priorities ^87^.

Research resource sharing should be bidirectional and ought not preclude LMIC access to data and specimens. Investment in equalizing these resources between research partners – including information technology and biobanking support – is needed as a part of the partnership plan and funding. This is particularly relevant with growing calls for universally available research data repositories and specimen biobanks to promote and replicate medical discoveries on a global scale ^88, 89^. While this has the potential to contribute to advancement of multiple research agendas, there are significant ethical tensions around informed consent, data security, return of results, and governance of unknown downstream research activities. Guidance on scientific data management and stewardship is available, for example the FAIR Guiding Principles ^90^, although these recommendations do not comment specifically on who has, or should have, data access. The International Network for the Demographic Evaluation of Populations and Their Health (INDEPTH) ^91^ serves as an example of one current LMIC-led data repository for longitudinal demographic health data in 52 countries. Access is provided to contributing institutions, and support teams based in South Africa and India are available to assist with technological barriers and formatting study data for the repository to offset this burden from study staff and investigators.

There are many potential benefits in sharing health research data and biologic specimens. This needs to be balanced, however, with careful attention to who ‘owns’ and ‘allows others’ to access these data and specimen repositories and whether groups that govern this information, which are most frequently based in HICs, gain a disproportionate amount of power in the form of knowledge production and distribution. If LMIC researchers and policy makers are dependent on the HIC groups to access data sets relevant to their population’s health, this can set back goals of developing robust, integrated national health information systems. It also has the potential to propagate use of health metrics and indicators designated as important by others, which may not necessarily reflect local priorities ^92^.

In adaptive platform clinical trials, post-trial access to interventions and biospecimens can be complicated by the dynamic nature of the research design as the final intervention or research population is not always the same as when the trial started. In such a context, consideration of post-trial access is needed not only during the initial planning stages, but also when significant modifications to the interventions or research protocol are made. This ensures ongoing transparency and realistic expectations about the translation of results.

### Knowledge translation

The impact of health research is minimal unless it is translated into clinical practice, health systems, or health policy. Efforts by all partners need to be made to translate research results into products and languages that are relevant to and can be understood by health system leadership and staff, research participants, local communities, and health policy personnel. This includes a need to prevent miscommunication about research results and rectify misinformation if it arises. The role and influence of LMIC partners is critical to provide guidance on optimal methods to disseminate research and incorporate findings into local practice and cultural contexts. Consideration around this transition needs to start as the research agenda is being set, given that research that is responsive to local priorities is most valuable to policy and practice. Involvement of key policy and practice stakeholders from study initiation through publication and dissemination can improve the likelihood of uptake, especially if those individuals are also in positions to directly apply the results of the research ^57^. While scientific validity and social value are traditionally heralded as primary influencers over the translation of scientific knowledge from research into practice, in many ways the uptake of research is also dependent on the structure, dynamics, and central LMIC inclusion within the research partnership.

For trials using adaptive platform trial designs, clear explanations for how the research design may have impacted interpretation of the results should be available for clinicians and other key stakeholders to accurately interpret findings. Lack of familiarity with the methodology of these trials may result in confusion during their uptake. Within an epidemic or pandemic context where research findings may be rapidly implemented, this is especially pertinent. Trial partnerships should prioritize effective communication of areas where there is confidence and areas where there may be variation in interpretations, including differing or evolving opinions about how to interpret the data and statistical analyses. The Ebola ça Suffit ring vaccination trial is an example. This was a stepwise cluster-randomized vaccine trial initiated in response to the Ebola outbreak in West Africa between 2014–2016. The methodology of using ring vaccination was actively debated ^93^ based on its deviation from using placebo-based control groups. The initial study reported a statistically significant estimate of 100% protection from Ebola among individuals within the clusters ^94^. Subsequently, a committee formed by the U.S. National Academies of Sciences, Engineering, and Medicine challenged this result using an intention-to-treat model that demonstrated a much lower vaccine efficacy rate of 65%, which did not reach statistical significance ^95^. These are arguably clinically- and policy-relevant discrepancies in how to interpret the same set of data. Issues like these differences need to be incorporated into discussions about policy and practice translation and doing so requires a firm foundational knowledge of how trials are designed.

### Research capacity strengthening and maintenance

Equity within transnational research partnerships requires dedicated attention to capacity strengthening to support sustainability of methodological and other research capabilities. While relevant and important for all partners to enhance their research skills and experience, the greater academic, institutional, and economic resources at the disposal of HIC researchers imply the need for research capacity efforts to primarily focus on the needs of LMIC partners. This should begin with a series of consultations to identify needs and continue through all research-related and post-trial partnership activities. This goal may be facilitated by the fact that competitive global health funding mechanisms are increasingly requiring research capacity strengthening as part of funding applications ^96^. When feasible, dedicated measures towards research capacity strengthening, including research and ethics training, mentorship, statistics and epidemiology guidance, and academic writing workshops, should be implemented in parallel with traditional research procedures.

Careful attention to each partner’s strengths, capabilities and needs is important to the productive identification of capacity strengthening opportunities that are mutually embraced, and potentially even mutually beneficial ^97^. Capacity strengthening that is targeted to specific needs is far more impactful than generic approaches, and it can take time to build relationships of trust that support meaningful building of knowledge and skills. Similarly, the capacity to translate research findings into policy and practice is felt to increase as programs and partnerships mature and gain experience over time ^98^.

 The lack of established research infrastructure and capacity to support rapid expansion of complex clinical trials has been a central barrier to LMIC participation in large adaptive platform trials early in the COVID-19 pandemic ^9^. Participation of the few LMIC sites that were able to engage was largely attributable to the presence of similar existing research and clinical infrastructure ^48^. As demonstrated by the collaboration between REMAP-CAP and CCA, leveraging existing research partnerships and relationships can be vitally important as systems strengthen. This includes programs like the African coaLition [sic] for Epidemic Research, Response, and Training (ALERRT) ^99^ and the Pan-African Network for Rapid Research, Response, Relief, and Preparedness for Infectious Diseases Epidemics (PANDORA). Both programs are transnational research consortiums with clinical trial experience and established networks that were essential to the African response to COVID-19.

In many ways the sizable time and financial investment plus the longitudinal nature of many adaptive platform clinical trials can be synergistic with research capacity strengthening efforts. These types of trials are able to ensure the long-standing involvement of LMIC research staff and academic partners while also providing opportunities for gaining experience and expertise ^10^. Because of the size and scope, large multi-national adaptive platform trial protocols and networks are well-positioned to “hibernate” during non-epidemic and -pandemic times but remain available for activation should conditions change ^100^. These periods of latency are opportune for dedicated investment towards building research capacity and partnership equity that may be deprioritized during an acute pandemic response.

## Conclusions

 Adaptive platform trials have risen in prominence globally in response to COVID-19, but the full potential, sustainability, and impact on LMIC research infrastructure remains to be seen. These trials are often implemented by transnational research partnerships, and these brings with them a host of equity considerations rooted in power and global resource asymmetries between partners from LMICs and HICs. While most aspects of transnational research partnership equity transcend specific research methodologies deployed by a partnership, the expansion of adaptive platform trials does highlight particular challenges. This includes awareness of fairness and influence as it pertains to global concentration of knowledge acquisition and study design experience, balancing universal study protocols with pluralistic research community values and objectives, and the potential impact longitudinal, high-investment research studies can have on strengthening and sustaining LMIC research capacity. Adaptive platform trials provide a distinctive opportunity for further empirical investigation and practice innovation to begin to narrow some of the inequities that exist between LMIC and HIC collaborative partners engaged in global health research.

## Data Availability

All data underlying the results are available as part of the article and no additional source data are required.
