# Supplementary material for: Towards achieving transnational research partnership equity: lessons from implementing adaptive platform trials in low- and middle-income countries
Source: Wellcome Open Res. 2023 Dec 6;8:120. Originally published 2023 Mar 16. [Version 2] doi: 10.12688/wellcomeopenres.18915.2 (PMC10714106; doi:10.12688/wellcomeopenres.18915.2)
Supplement: Peer review history

# Reviewer report: Reviewer response for version 2

**DOI:** 10.21956/wellcomeopenres.22673.r70991

**Published:** 2023-12-11

**Authors:** Thomas Nyirenda

**Affiliations:** 1 European and Developing Countries Clinical Trials Partnership, University of Stellenbosch Global Health Department, Cape Town, South Africa

I am happy with the authors’ comments and amendments following my input.

Is the work clearly and accurately presented and does it cite the current literature?

Partly

If applicable, is the statistical analysis and its interpretation appropriate?

Not applicable

Are all the source data underlying the results available to ensure full reproducibility?

No source data required

Is the study design appropriate and is the work technically sound?

Yes

Are the conclusions drawn adequately supported by the results?

Yes

Are sufficient details of methods and analysis provided to allow replication by others?

Yes

Reviewer Expertise:

NA

I confirm that I have read this submission and believe that I have an appropriate level of expertise to confirm that it is of an acceptable scientific standard.

# Reviewer report: Reviewer response for version 1

**DOI:** 10.21956/wellcomeopenres.20973.r62072

**Published:** 2023-08-14

**Authors:** Peter van der Graaf

**Affiliations:** 1 Northumbria University, Newcastle upon Tyne, UK

A well written and thoughtful paper with a clear conclusion on applying equity principles in transnational partnerships from the perspective of low and middle-income countries when collaborating on adaptive platform trials. I enjoyed reading the paper.

 The authors could strengthen the paper by adding more examples to each theme, particularly around practical solutions suggested in response to identified challenges. It is also not always clear to me what the unique dimensions of the issues are highlighted under each theme for adaptive platform trial designs. The authors state that these challenges are heightened for this type of design, but do not always make clear in what ways.

 For example, under the ethics oversight theme (page 8, 3 ^rd^ paragraph), the authors argue that there is a need for more familiarity with adaptive platform trial designs among RECs and DMCs, including unique ethical considerations of these different methodologies. What are these ethical considerations and how are they unique?

 Similarly, under the theme of regulatory oversight, (page 8, paragraph 5), the authors recommend re-evaluating processes for recognising the legitimacy of alternative regulatory frameworks. Can they give an example of such a framework? I appreciate they added a reference, but this does not give much insight; showing a brief practical example of how to apply an alternative framework to adaptive platform trials would be more helpful.

 In the next paragraph, the authors identify a need for LMIC-specific guidance for internationally funded adaptive trials. What should this specific guidance look like and how will it differ from existing FDA and EMA guidance, as mentioned by the authors, to incorporate the experiences of regulatory bodies in LMICs?   

 Another example, when describing the challenges of post-trial access (PTA) to interventions, data, and specimens, the authors state that “providing PTA may be more realistic for some types of research interventions and partnerships than others” (page 9, 4 ^th^ paragraph). What type of research interventions and partnerships are more amenable for PTA, given the challenges for LMIC partners to access this data?

 I would also have liked to see a bit more detail on how the themes were developed. The authors briefly describe the process (triangulation of insights from the rapid narrative review with unpublished experiences and practices from meetings with project participants, and critical review by experts), but do not show how initial themes from the review were revised based on the meetings and expert reviews.  It would be insightful to learn how the authors managed the blending of these different types of knowledge into the eight themes.

 Two of the current themes appear to be more cross-cutting than the others and the authors might want to consider incorporating these themes in the other themes or making clearer the link between these two themes and the other ones.

 For example, the knowledge translation theme seems to be dealing with the variation in interpretations of results through effective communication and preventing miscommunication. This is quite a limited take on knowledge translation with a focus on dissemination through appropriate language and products. However, the authors also mention the early involvement of key policy and practice stakeholders, which links to earlier suggestions in other themes to engage these groups in the trial design and conduct, which widens the scope of knowledge translational activities and makes it more a cross-cutting theme. (E.g., how to involve key stakeholders in all stages in response to issues identified under each theme to incorporate research results into local practice and cultural contexts?).  This would also help to provide more clarity on the suggestion made by the authors at the end of this theme “to incorporate these uses into discussions about policy and practice translations” How? (page 10, paragraph 2).  

 The second cross-cutting theme could be research capacity strengthening and maintenance. Building the research skills and experience of LMIC partners is mentioned in several of the other themes (for example, when developing epistemic structures and ethics oversight) and these suggestions could be linked together to illustrate the difference dimensions of capacity building required in transnational research partnerships for adaptive platform trial designs. What I feel is currently missing in this theme is a wider reflection on the capacity building needs for HIC researchers, as the current theme suggest that capacity building is particularly required for LMIC partners. How can HIC researchers be trained in getting a better understanding of LIMC needs, unique knowledge and local contexts, and upskilled in their understanding of adaptive platform trials to support transnational partnerships and ensure they are equitable?   

 I hope these suggestions are helpful and look forward to the authors’ responses to my comments.

Is the work clearly and accurately presented and does it cite the current literature?

Yes

If applicable, is the statistical analysis and its interpretation appropriate?

Not applicable

Are all the source data underlying the results available to ensure full reproducibility?

Yes

Is the study design appropriate and is the work technically sound?

Yes

Are the conclusions drawn adequately supported by the results?

Yes

Are sufficient details of methods and analysis provided to allow replication by others?

No

Reviewer Expertise:

Public Health, Knowledge Mobilisation, Collaborative Research Partnerships

I confirm that I have read this submission and believe that I have an appropriate level of expertise to confirm that it is of an acceptable scientific standard, however I have significant reservations, as outlined above.

# Response

**Published:** 2023-11-11

**Authors:** Chelsea Modlin

**Affiliations:** Johns Hopkins Medicine, USA

Thank you for the opportunity to submit a revised version of the manuscript **“Towards achieving transnational research partnership equity: lessons from implementing adaptive platform trials in low- and middle-income countries”** (manuscript 18915) *. *We appreciate the time and attention dedicated to improving the manuscript and have incorporated the suggestions made by the reviewer. Please see below for a point-by-point response to the reviewer comments.  

**Comment 1: **The authors could strengthen the paper by adding more examples to each theme, particularly around practical solutions suggested in response to identified challenges. It is also not always clear to me what the unique dimensions of the issues are highlighted under each theme for adaptive platform trial designs. The authors state that these challenges are heightened for this type of design, but do not always make clear in what ways. For example, under the ethics oversight theme (page 8, 3rd paragraph), the authors argue that there is a need for more familiarity with adaptive platform trial designs among RECs and DMCs, including unique ethical considerations of these different methodologies. What are these ethical considerations and how are they unique?

Response: We appreciate the focus on this issue and will reference back to the response to Reviewer 2 Comment 4 as the ethical considerations unique to adaptive platform trials was explored in detail within another paper included in the same series as our manuscript.  

**Comment 2: **Similarly, under the theme of regulatory oversight, (page 8, paragraph 5), the authors recommend re-evaluating processes for recognising the legitimacy of alternative regulatory frameworks. Can they give an example of such a framework? I appreciate they added a reference, but this does not give much insight; showing a brief practical example of how to apply an alternative framework to adaptive platform trials would be more helpful.

Response: This is a great point. For the sake of word count consolidation and to avoid redundancy with work that was completed by others within the broader project, we have added a reference to where readers can find a more detailed discussion of current and alternative regulatory frameworks for adaptive platform trials ( doi.org/10.12688/wellcomeopenres.19058.1). Page 15, Paragraph 1: “A more detailed discussion of regulatory considerations for adaptive platform trials can be found in another article in this series (84).”  

**Comment 3: **In the next paragraph, the authors identify a need for LMIC-specific guidance for internationally funded adaptive trials. What should this specific guidance look like and how will it differ from existing FDA and EMA guidance, as mentioned by the authors, to incorporate the experiences of regulatory bodies in LMICs?

Response: The suggestion raised here is also important and aligned with the purpose of our manuscript compared to the reference cited in the response to Comment 2. We have added the following text to serve as one hypothetical example given a dearth of real-world examples within the published literature. Follow up work qualitatively describing these types of details within multinational adaptive platform design trials in LMICs during COVID-19 is one of the projects that is currently being planned as a result of this preliminary work. Pages 15, Paragraph 1: “In addition, although basic regulatory and design requirements for clinical trials using adaptive methods are available from the U.S. Food and Drug Administration (FDA) (82) and the European Medicines Agency (EMA) (83), these are recommendations based on experiences and regulatory bodies situated in HICs. For example, the FDA report Adaptive Designs for Clinical Trials of Drugs and Biologics Guidance for Industry (82) details the complexities of planning for simulations to reduce statistical errors that are specific to potential components of adaptive platform trials such as adaptations to treatment arm interventions or evolving endpoints. It is possible that expertise in these advanced statistical methods is limited in LMICs and other under-resourced areas and there is no formal guidance for how to develop this skill set where it did not previously exist, leaving it up to the discretion of individuals partnerships. While this may not necessarily be within the scope of the FDA to determine, special regulatory considerations for internationally funded adaptive trials conducted in LMICs, and LMIC-specific guidance for these trials, including capacity development recommendations or requirements, is needed.”  

**Comment 4: **Another example, when describing the challenges of post-trial access (PTA) to interventions, data, and specimens, the authors state that “providing PTA may be more realistic for some types of research interventions and partnerships than others” (page 9, 4th paragraph). What type of research interventions and partnerships are more amenable for PTA, given the challenges for LMIC partners to access this data?

Response: We have added an example to the text specific to COVID-19 multinational adaptive trials based on the discussion that was held with the REMAP-CAP group. Page 16-17, Paragraph 1: “In adaptive platform clinical trials, PTA to successful study-related interventions and biospecimens can be complicated by the dynamic nature of the research design as the final intervention or research population is not always the same as when the trial started. COVID-19 adaptive platform trials included widely-available therapeutics (e.g., aspirin) and novel therapeutics (e.g., monoclonal antibodies) at various points of time during the pandemic. Due to a combination of limited global availability and stipulations of the pharmaceutical companies sponsoring these trials, it was often not realistic to expect novel therapeutics to be available post-trial in LMICs (9). Intervention arms therefore focused on treatment options that would be locally feasible to implement, which were often repurposed medications.”  

**Comment 5: **I would also have liked to see a bit more detail on how the themes were developed. The authors briefly describe the process (triangulation of insights from the rapid narrative review with unpublished experiences and practices from meetings with project participants, and critical review by experts), but do not show how initial themes from the review were revised based on the meetings and expert reviews.  It would be insightful to learn how the authors managed the blending of these different types of knowledge into the eight themes.

Response: We have addended the methods to include more detail as suggested by the reviewer. Page 10 , Paragraph 3: “Notes taken from this meeting were integrated into the working themes identified from the literature reviews (including areas of congruence and  conflict),  discussed between the authors until consensus was reached, and a preliminary report was drafted. This report was then discussed during a project group meeting in Geneva, Switzerland in July 2022. Findings were iteratively revised based on the discussion and feedback from this meeting, including areas relevant to the scope of this report compared to other aims of the project, and from critical review by experts in ethics, global health, and adaptive clinical trial methods who provided staged feedback on preliminary drafts of this report.”  

**Comment 6: **Two of the current themes appear to be more cross-cutting than the others and the authors might want to consider incorporating these themes in the other themes or making clearer the link between these two themes and the other ones. For example, the knowledge translation theme seems to be dealing with the variation in interpretations of results through effective communication and preventing miscommunication. This is quite a limited take on knowledge translation with a focus on dissemination through appropriate language and products. However, the authors also mention the early involvement of key policy and practice stakeholders, which links to earlier suggestions in other themes to engage these groups in the trial design and conduct, which widens the scope of knowledge translational activities and makes it more a cross-cutting theme. (E.g., how to involve key stakeholders in all stages in response to issues identified under each theme to incorporate research results into local practice and cultural contexts?).  This would also help to provide more clarity on the suggestion made by the authors at the end of this theme “to incorporate these uses into discussions about policy and practice translations” How? (page 10, paragraph 2).

Response: The is an important point and one of the inherent challenges within the international research partnership equity space, namely that many of these themes overlap, or sometimes even contradict, one another depending on the specific context they are being applied to. We have renamed this theme ‘Knowledge Translation and Dissemination’ in an attempt to distinguish that here we primarily focus on the transformation of scientific knowledge generated by a research partnership to both the local community and scientific community at large. We have also added the following text to the Discussion section to help point out that none of these themes should be treated as siloed from one another given the dynamic and often individualized approach required to meet certain equity strengths, limitations, and goals within a research partnership, geographic setting, research focus, etc. Page 17, Paragraph 3: “Issues like these differences need to be incorporated into discussions about policy and practice translation by inviting pluralistic analyses and interpretations of research data and conclusions. It is particularly important to include those with expertise in local contexts, and what these differences would mean in terms of health policy actions taken in response to this new scientific knowledge. Doing so requires a firm foundational knowledge of how trials are designed and associated limitations of specific methodologies.” Page 19, Paragraph 4: “We have presented eight themes that emerged from the literature and recent experience, but it is important to note that none of these topics occur as siloed entities from one another. There is significant overlap in many of the themes, for example between regulatory oversight and PTA when sponsors directly control the availability of interventions, data and biospecimens; or between research capacity building and essentially all of the other themes. Enhanced LMIC administrative, ethics, scientific, infrastructure, and institutional capacities would  help support and inform LMIC representation in the other seven themes discussed.”  

**Comment 7: **The second cross-cutting theme could be research capacity strengthening and maintenance. Building the research skills and experience of LMIC partners is mentioned in several of the other themes (for example, when developing epistemic structures and ethics oversight) and these suggestions could be linked together to illustrate the difference dimensions of capacity building required in transnational research partnerships for adaptive platform trial designs. What I feel is currently missing in this theme is a wider reflection on the capacity building needs for HIC researchers, as the current theme suggest that capacity building is particularly required for LMIC partners. How can HIC researchers be trained in getting a better understanding of LIMC needs, unique knowledge and local contexts, and upskilled in their understanding of adaptive platform trials to support transnational partnerships and ensure they are equitable? 

Response: This is a very key point. We have addressed the bilateral nature of capacity building with the following text and have also added text to the Discussion section mentioned in Comment 6 to highlight that these themes intersect with one another. Page 18, Paragraph 1: “…focusing research capacity efforts on the LMIC partners. This does not preclude the importance of HIC capacity building, particularly in understanding LMIC contexts and needs as well as evaluating internal power dynamics to determine how HIC-based institutions, researchers, and sponsors can initiate or transition into positions of allyship instead of leadership.”   We greatly appreciate the careful consideration of our work and thank the reviewer for offering the opportunity to strengthen the manuscript based on these helpful comments.

# Reviewer report: Reviewer response for version 1

**DOI:** 10.21956/wellcomeopenres.20973.r59883

**Published:** 2023-08-02

**Authors:** Thomas Nyirenda

**Affiliations:** 1 European and Developing Countries Clinical Trials Partnership, University of Stellenbosch Global Health Department, Cape Town, South Africa

1. There is a generalisation that adaptive trial platforms were poorly understood before COVID-19 pandemic. These platforms have been used in NCD research especially in Cancer. PANACEA ( PanACEA (panacea-tb.net)) infectious disease (TB) research consortium funded by EDCTP has also been in existence since 2007. These two aspects are worth mentioning in the paper.
2. In relation to PANACEA one of the lessons that be requested for sharing from them are lessons (if any) around challenges of transferring leadership from the scientists in the north to those in the south during the course of long research programs
3. Under ethics one of the recent projects worth mentioning is the PREPARED which aims to build global consensus in making research during crises ethical ( PREPARED (prepared-project.eu)
4. Adaptive trial designs are indeed very complex and risk wastage of invested money and time if they end up being poorly designed and fail to answer the questions they were designed to address. The risk rises as during the course of the trials endpoints constantly change too. The Bill and Melinda Gates Foundation have funded a Design, Analyse and Communicate Programme (recently launched in Africa in Cameroon as its host country) - Welcome • DAC Trials (tghn.org). This great initiative deserves a mention in the paper for the quality and efficiency reasons mentioned. 

Is the work clearly and accurately presented and does it cite the current literature?

Partly

If applicable, is the statistical analysis and its interpretation appropriate?

Not applicable

Are all the source data underlying the results available to ensure full reproducibility?

No source data required

Is the study design appropriate and is the work technically sound?

Yes

Are the conclusions drawn adequately supported by the results?

Yes

Are sufficient details of methods and analysis provided to allow replication by others?

Yes

Reviewer Expertise:

Clinical research/ trials and capacity development

I confirm that I have read this submission and believe that I have an appropriate level of expertise to confirm that it is of an acceptable scientific standard, however I have significant reservations, as outlined above.

# Response

**Published:** 2023-11-11

**Authors:** Chelsea Modlin

**Affiliations:** Johns Hopkins Medicine, USA

Thank you for the opportunity to submit a revised version of the manuscript **“Towards achieving transnational research partnership equity: lessons from implementing adaptive platform trials in low- and middle-income countries”** (manuscript 18915) *. *We appreciate the time and attention dedicated to improving the manuscript and have incorporated the suggestions made by the reviewer. Please see below for a point-by-point response to the reviewer comments.  

**Comment 1: **There is a generalisation that adaptive trial platforms were poorly understood before COVID-19 pandemic. These platforms have been used in NCD research especially in Cancer. PANACEA ( PanACEA (panacea-tb.net)) infectious disease (TB) research consortium funded by EDCTP has also been in existence since 2007. These two aspects are worth mentioning in the paper.

Response: We appreciate the review’s insights into this topic and have added the suggested resources into the introduction of the manuscript which is also linked to the PanACEA website for reference. Page 4, Paragraph 1: “These types of trial design were already familiar to some endemic infectious disease research (e.g.,  the PanACEA tuberculosis trials) and non-communicable diseases research, particularly in oncology. However, increased attention to adaptive platform trials arose  in response to recent global infectious epidemics and pandemics.”  

**Comment 2: **In relation to PANACEA one of the lessons that be requested for sharing from them are lessons (if any) around challenges of transferring leadership from the scientists in the north to those in the south during the course of long research programs.

Response: Thank you for this note – the transfer of leadership from HICs to LMICs is a component of leadership considerations and power differentials within international research partnerships. We have added the following text to highlight this issue although our review of adaptive platform trials did not specifically identify this theme. It suggests the importance of further qualitative investigation with researchers who participate in multinational adaptive platform trials to elucidate how this consideration transferred to adaptive trials specifically. Page 15, Paragraph 2: “For longitudinal research programs, the transfer of leadership from HIC- to LMIC-based researchers is often advocated for but can, in reality, sometimes be hindered by institutional and individual-level factors. But this is not always the case and there are many examples of partnerships that  have enacted very intentional efforts in the training of LMIC leaders and transfer of partnership responsibilities.”  

**Comment 3: **Under ethics one of the recent projects worth mentioning is the PREPARED which aims to build global consensus in making research during crises ethical ( PREPARED (prepared-project.eu

Response: This suggestion has been added to the ‘Ethics Oversight’ row under ‘Potential Solutions’ in Table 2 with a link to the PREPARED initiative website for reference. Table 2: “Ethical oversight during public health emergencies that goes beyond IRBs is also developing, including efforts such as the PREPARED initiative.”  

**Comment 4: **Adaptive trial designs are indeed very complex and risk wastage of invested money and time if they end up being poorly designed and fail to answer the questions they were designed to address. The risk rises as during the course of the trials endpoints constantly change too. The Bill and Melinda Gates Foundation have funded a Design, Analyse and Communicate Programme (recently launched in Africa in Cameroon as its host country) - Welcome • DAC Trials (tghn.org). This great initiative deserves a mention in the paper for the quality and efficiency reasons mentioned.

Response: This is a very relevant consideration on the ethical management of adaptive platform trials especially as there is debate as to whether the cost of adaptive trials exceeds that of large-scale fixed trial designs. It overlaps with the discussion in the paper “Adaptive clinical trials in public health emergency contexts: ethics considerations” ( doi.org/10.12688/wellcomeopenres.19057.1) which is a part of the same series as our manuscript. We have added the following text to help highlight the connection between the two papers: Page 14, Paragraph 1: “Another article in this series provides additional analyses of the ethical considerations unique to adaptive platform trials (75).”   We greatly appreciate the careful consideration of our work and thank the reviewer for offering the opportunity to strengthen the manuscript based on these helpful comments.

# Reviewer report: Reviewer response for version 1

**DOI:** 10.21956/wellcomeopenres.20973.r59891

**Published:** 2023-08-02

**Authors:** Justin Pulford

**Affiliations:** 1 Liverpool School of Tropical Medicine, Liverpool, UK

This paper presents a synthesis of equity issues common to transnational research partnerships and then examines these issues from the point of view of adaptive platform trials. Potential solutions to addressing identified inequities, especially opportunities where adaptive platform trials may be especially well suited to addressing inequities, are identified. Suggested amendments to the paper include:

**Introduction**

 Given the focus on equity, it would be helpful to include a working definition somewhere in the introduction.

 Amend the sentence: ‘We offer a thematic analysis of eight areas where asymmetries exist…’ to ‘We offer a thematic analysis of eight areas where asymmetries **can **exist…’

**Methods**

 While I recognise the authors employed both ‘rapid’ and ‘selective’ reviews, it would still be informative to understand how much literature was identified and subsequently utilised (and what type of literature etc).  The authors could consider reporting selected PRISMA criteria as appropriate.

 The list of publications subsequently included in both reviews could be presented as a supplementary file.

 Given the focus on equity, it would be useful to include some description of the authors’ LMIC representation and North-South research partnership experience in the methods.

**Results**

 There is a lot of content under each theme and it is not always presented in a consistent way across themes. In general, there seems to be an overview of challenges related to the theme, some proposed solutions that are not necessarily specific to adaptive platform trials and then considerations/recommendations that are specific to adaptive platform trails.  In some cases an example is presented, in some cases not. I would suggest employing a consistent format throughout and include (consistent) sub-headings. The text under each theme is quite lengthy, so consideration could be given to the use of a Table, e.g. Each theme could begin with a description of the equity issues identified and then proposed solutions (both general and adaptive platform specific) could be presented in an overarching (covering all 8 themes) table. This would make it easier to follow and would better highlight the adaptive platform-specific content.

 Where recommendations are made, it is not always clear who the intended audience is. This needs to be made clearer. If you adopt the tabular approach suggested above, then against each stated recommendation you could identify the intended audience/s.

**Conclusion**

 If the authors reduce text in the ‘results’ section as suggested, then this would create word space for a more traditional ‘discussion then conclusion’ format.  I suggest this as the current conclusion touches on two key discussion points that warrant a stronger connection with the equity literature: 1) equity issues that appear common across research methodologies; and 2) equity issues that are especially pertinent in an adaptive platform trial context.  The latter, in particular, warrants elaboration as this is a key contribution of the paper, i.e. what common equity issues are adaptive platform trials especially well placed to address, who should take responsibility and how.

Is the work clearly and accurately presented and does it cite the current literature?

Yes

If applicable, is the statistical analysis and its interpretation appropriate?

Not applicable

Are all the source data underlying the results available to ensure full reproducibility?

Partly

Is the study design appropriate and is the work technically sound?

Yes

Are the conclusions drawn adequately supported by the results?

Yes

Are sufficient details of methods and analysis provided to allow replication by others?

Partly

Reviewer Expertise:

Research capacity strengthening theory, methods and evaluation

I confirm that I have read this submission and believe that I have an appropriate level of expertise to confirm that it is of an acceptable scientific standard, however I have significant reservations, as outlined above.

# Response

**Published:** 2023-11-11

**Authors:** Chelsea Modlin

**Affiliations:** Johns Hopkins Medicine, USA

Thank you for the opportunity to submit a revised version of the manuscript **“Towards achieving transnational research partnership equity: lessons from implementing adaptive platform trials in low- and middle-income countries”** (manuscript 18915) *. *We appreciate the time and attention dedicated to improving the manuscript and have incorporated the suggestions made by the reviewer. Please see below for a point-by-point response to the reviewer comments.  

**Comment 1: **Given the focus on equity, it would be helpful to include a working definition somewhere in the introduction.

Response: Thank you for the suggestion. We have added a definition of research partnership equity to the introduction. Page 4, Paragraph 2: “We define “research partnership equity” as collaborative and inclusive research practices that incorporate fairness of opportunity and prioritize mutually beneficial inputs, processes, outputs, and impact.”  

**Comment 2: **Amend the sentence: ‘We offer a thematic analysis of eight areas where asymmetries exist…’ to ‘We offer a thematic analysis of eight areas where asymmetries **can** exist…’

Response: This has been revised. Page 6, Paragraph 3: “We offer a thematic analysis of eight areas where asymmetries can exist within transnational research partnerships…”  

**Comment 3: **While I recognise the authors employed both ‘rapid’ and ‘selective’ reviews, it would still be informative to understand how much literature was identified and subsequently utilised (and what type of literature etc).  The authors could consider reporting selected PRISMA criteria as appropriate.

Response: We appreciate the response and do recognize the importance of systematic literature review to decrease the risk of bias within the analysis. We have revised the methods section to include the inclusion criteria utilized and added limitations, including how this may have impacted our conclusions and replicability. Page 10, Paragraphs 1 & 2: “We performed a rapid narrative review (49) on transnational research partnership equity, which was cross-referenced with selective reviews of literature on adaptive clinical trial designs and adaptive platform designs, including those reporting COVID-19 and Ebola research in order the answer the following question: What transnational research partnership equity considerations are relevant to adaptive platform clinical trials? This approach allowed for broad examination of emerging evidence, evaluation for more specific lines of inquiry, clarification of concepts, and synthesis of two largely separate, but converging lines of published literature. PubMed/MEDLINE, Scopus, and Google Scholar databases were reviewed in March and revisited in May of 2022 using keyword searches for ‘equity’ OR ‘fairness’ AND ‘international research partnerships’ OR ‘global research partnerships’ ‘OR ‘transnational research partnerships’ OR ‘North-South partnerships.’ Eligibility criteria included postings that 1.) describing or categorizing the components of international research partnership equity; 2.) were peer-reviewed and featured primary empirical research, published guidelines or policies, program reports, normative/conceptual articles, or commentaries/editorials; and 3.) published in English. There were no publication date specifications. The selective literature search on adaptive clinical trials focused on ethical analyses and operationalization of these trials and included search terms ‘adaptive clinical trial +/- designs’ OR ‘platform trial’ AND ‘ethics’ OR ‘ethical considerations’ OR ‘experience’ OR ‘operationalization’ OR ‘implementation.’ Eligibility criteria were identical to those listed above. The citations were appraised and relevant postings  were included in subsequent abstract and full-text reviews. Searches were conducted by two independent reviewers who met  to discuss findings and develop and apply thematic categorizations. Any variation in interpretation or classification of findings was resolved by consensus. Because of the limited project timeframe, further systematic tracking of the literature aside was not pursued. One of the limitations of this approach is that we cannot exclude any potential bias introduced from an informal review as opposed to using a more formal approach, perhaps limiting the replicability of our findings. Additionally, the definition and components of research partnership equity are evolving over time complicating literature identification.  

**Comment 4: **The list of publications subsequently included in both reviews could be presented as a supplementary file.

Response: Please see response to Comment 3.  

**Comment 5: **Given the focus on equity, it would be useful to include some description of the authors’ LMIC representation and North-South research partnership experience in the methods.

Response: We have included the suggested description but included this as a reflexivity statement. Page 19, Paragraph 2: “J.A. and C.M. were the researchers approached by the WHO Ethics and Governance Unit to lead this project, which is one of five sub-aims discussed during the project group meeting in Geneva, Switzerland in July 2022. The authors of this manuscript are a combination of HIC- and LMIC-based researchers all of whom have experience working in LMIC/HIC international research collaborations, and with interrogating the ethics and regulatory dimensions of adaptive and alternative trial methods. LMIC-based authors G.C., W.N., and J.T. held the same roles and responsibilities as HIC-based authors J.S. and N.K., primarily providing critical feedback on preliminary and subsequent drafts of the manuscript.”  

**Comment 6: **There is a lot of content under each theme and it is not always presented in a consistent way across themes. In general, there seems to be an overview of challenges related to the theme, some proposed solutions that are not necessarily specific to adaptive platform trials and then considerations/recommendations that are specific to adaptive platform trails.  In some cases an example is presented, in some cases not. I would suggest employing a consistent format throughout and include (consistent) sub-headings. The text under each theme is quite lengthy, so consideration could be given to the use of a Table, e.g. Each theme could begin with a description of the equity issues identified and then proposed solutions (both general and adaptive platform specific) could be presented in an overarching (covering all 8 themes) table. This would make it easier to follow and would better highlight the adaptive platform-specific content.

Response: Following the reviewer’s suggestion we have substantially revised the manuscript format and Table 2. Each of the eight themes is now in sections with headings ‘Challenges’ (general to international research partnerships) and ‘Considerations for Adaptive Platform Trials’. In-depth examples from the text have been moved to separate boxes to help distinguish these from the main text. Suggested solutions from the literature review have been moved to an expanded version of Table 2.  

**Comment 7: **Where recommendations are made, it is not always clear who the intended audience is. This needs to be made clearer. If you adopt the tabular approach suggested above, then against each stated recommendation you could identify the intended audience/s.

Response: Thank you for pointing this out. We have updated the text to reflect the intended audience identified for the project. Page 10, Paragraph 1: “The intended audiences of this project are researchers, institutions, funders and sponsors who currently or plan to engage in multinational adaptive platform clinical trials.”  

**Comment 8: **If the authors reduce text in the ‘results’ section as suggested, then this would create word space for a more traditional ‘discussion then conclusion’ format.  I suggest this as the current conclusion touches on two key discussion points that warrant a stronger connection with the equity literature: 1) equity issues that appear common across research methodologies; and 2) equity issues that are especially pertinent in an adaptive platform trial context.  The latter, in particular, warrants elaboration as this is a key contribution of the paper, i.e. what common equity issues are adaptive platform trials especially well placed to address, who should take responsibility and how.

Response: The suggested revisions have been made, including changing the ‘Findings’ section to ‘Results’ and including a short ‘Discussion’ section to summarize how adaptive platform trials can help inform international research partnership equity. This was kept relatively brief to avoid redundancy with the information reviewed in the results section which includes more detail. We greatly appreciate the careful consideration of our work and thank the reviewer for offering the opportunity to strengthen the manuscript based on these helpful comments.
